# Mechanical Small Bowel Obstruction Secondary to Low-Grade Appendiceal Mucinous Neoplasm: A Case Report

**DOI:** 10.7759/cureus.114182

**Published:** 2026-08-08

**Authors:** Santiago Andrés Rojas Guanoluisa, José Manuel García Romero, Elizabeth Alcalá Ochoa, Alejandro Morales Rubio, Yael Castaneda-Cardoso

**Affiliations:** 1 Surgery, Hospital General de Querétaro, Querétaro, MEX; 2 General Practice, Universidad Autónoma de Querétaro, Querétaro, MEX; 3 Plastic Surgery, University Hospital Coventry and Warwickshire, Coventry, GBR; 4 Vascular Surgery, Instituto Nacional de Cardiología Ignacio Chávez, Mexico City, MEX

**Keywords:** acute abdomen, appendiceal mucocele, intestinal obstruction, low-grade appendiceal mucinous neoplasm, pseudomyxoma peritonei

## Abstract

Low-grade appendiceal mucinous neoplasm (LAMN) is an uncommon epithelial tumor that is frequently diagnosed incidentally and only rarely produces mechanical small bowel obstruction, with previous reports limited to isolated cases. We describe a 53-year-old man with a four-day history of periumbilical pain, obstipation, nausea, and bilious vomiting. Examination showed abdominal distension, decreased bowel sounds, and rebound tenderness. Contrast-enhanced computed tomography (CT) demonstrated a 7 cm heterogeneous right hemiabdominal mass with pericecal fat stranding that favored, but did not definitively establish, an appendiceal origin, prompting emergency exploratory laparotomy. Intraoperatively, a markedly dilated, mucin-filled appendix was densely adherent to the terminal ileum, producing mechanical small bowel obstruction; careful adhesiolysis and en bloc appendectomy were performed without rupture of the lesion. Histopathology confirmed LAMN with negative circumferential and basal margins and no lymphovascular, perineural, or lamina propria invasion, staged as pTis(LAMN) per the American Joint Committee on Cancer (AJCC) eighth edition. The postoperative course was uneventful, with oral intake resumed at 48 hours and discharge on postoperative day 3. Surveillance included clinical review at two weeks and three months, with double-contrast CT planned at six months and joint follow-up with oncologic surgery. This case highlights dense adhesion to the terminal ileum, rather than perforation, torsion, or internal herniation, as a rare mechanism of LAMN-related obstruction, and reinforces that atraumatic surgical handling is essential to avoid mucin spillage and pseudomyxoma peritonei.

## Introduction

Appendiceal mucinous neoplasms are uncommon epithelial tumors defined by the accumulation of mucin within the appendiceal lumen, identified in approximately 1% of appendectomy specimens [1,2]. Among these, low-grade appendiceal mucinous neoplasms (LAMNs) are of particular clinical importance because they may remain silent until they precipitate a surgical emergency such as mechanical small bowel obstruction, and their presentation frequently mimics acute appendicitis, delaying recognition and appropriate surgical planning [3].

Appendiceal epithelial neoplasms are classified along a spectrum of increasing atypia and invasiveness: LAMN, high-grade appendiceal mucinous neoplasm (HAMN), and mucinous adenocarcinoma, the latter distinguished by unequivocal invasion beyond the muscularis mucosae [4]. LAMN is identified in approximately 0.2%-1.7% of appendectomy specimens, most often as an incidental finding during surgery for presumed acute appendicitis or on cross-sectional imaging obtained for unrelated indications [4,5]. Progressive mucin hypersecretion by a dysplastic but noninvasive epithelium distends the appendiceal lumen and thins the wall, producing a mucocele; the muscularis propria typically remains intact, the histological feature separating LAMN from invasive mucinous adenocarcinoma [4]. Because the clinical picture is nonspecific, the differential diagnosis of a periappendiceal mass with obstructive symptoms includes acute appendicitis, a nonneoplastic mucocele, cecal or ileocecal Crohn's disease, cecal volvulus, and, in women, an adnexal or ovarian mucinous tumor [1,6].

Mechanical small bowel obstruction is an exceptionally uncommon presentation of LAMN, reported almost exclusively as isolated cases resulting from adhesive band formation, internal herniation, or direct adhesion of the lesion to adjacent bowel [7-9]. This mechanism, external compression and fibrous adhesion rather than luminal invasion, has direct surgical implications, since preoperative recognition of a mucin-filled appendiceal mass allows the operating team to plan an en bloc, atraumatic resection from the outset rather than performing blind adhesiolysis that risks rupturing the lesion.

LAMN is histologically noninvasive but is not clinically inert: rupture of the mucin-filled lumen, whether spontaneous or iatrogenic, can seed mucinous epithelium into the peritoneal cavity and lead to pseudomyxoma peritonei, a condition with a markedly different prognosis and management pathway [5,10,11]. This risk is the principal rationale for the atraumatic surgical technique described in this report. To our knowledge, obstruction of the terminal ileum caused specifically by dense adhesion to an intact (nonruptured, nontorsed) LAMN, as opposed to perforation, torsion, or internal hernia, is rarely described, which is the specific novelty of the present case. By describing this atypical, obstructive presentation, we aim to provide surgical teams with practical insight into the diagnostic nuances and therapeutic decision-making required for managing this rare appendiceal pathology.

## Case presentation

A 53-year-old male patient with no significant chronic-degenerative history, aside from a right shoulder surgery performed two years prior, presented to the emergency department with a four-day history of progressively worsening periumbilical pain, nausea, and obstipation, which subsequently evolved into diffuse abdominal cramping accompanied by bilious emesis. On initial physical examination, the patient was alert and oriented, with tachycardia and mild diaphoresis. Abdominal evaluation revealed distension and diminished peristalsis, with a positive rebound tenderness sign; no masses were detected on abdominal palpation or digital rectal examination.

Laboratory studies revealed mild thrombocytopenia (127,000/µL; reference: 130,000-450,000/µL), a normal leukocyte count (9,750/µL; reference: 4,000-10,000/µL) with marked neutrophilia (82.4%; reference: 50%-65%) and relative lymphopenia (11.6%; reference: 20%-40%), and hyperglycemia (154 mg/dL; reference: 70-105 mg/dL). Hemoglobin (15.6 g/dL; reference: 13-18 g/dL), hematocrit (45.4%; reference: 40%-54%), and red cell indices (mean corpuscular volume (MCV): 86 fL; reference: 80-100; mean corpuscular hemoglobin (MCH): 29.5 pg; reference: 27-32) were within normal limits; eosinophils, basophils, and monocytes were within or near normal limits. C-reactive protein, serum electrolytes, creatinine, lactate, and tumor markers (CEA, CA-125) were not obtained.

**Figure 1:**
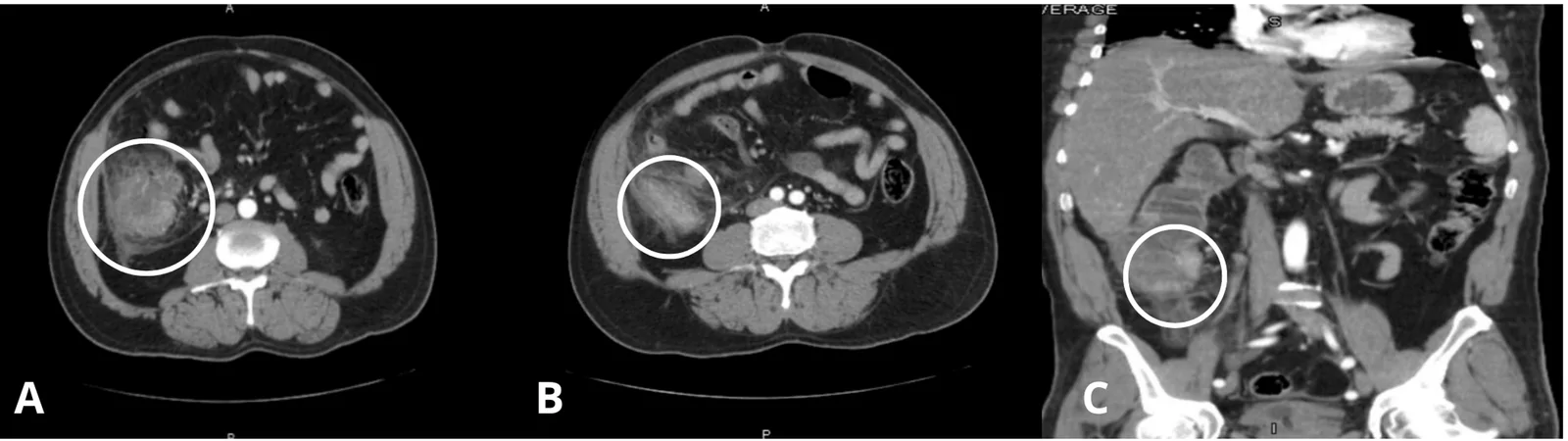
Contrast-enhanced abdominal CT showing the appendiceal mass at presentation CT: computed tomography CT demonstrating a heterogeneous 7 cm mass (white circle) in the right hemiabdomen with central hypoattenuation, irregular peripheral enhancement, and marked surrounding inflammatory fat stranding. (A, B) Axial views at the level of the cecum show the mass abutting the ascending colon/hepatic flexure and the second portion of the duodenum, although its exact organ of origin could not be definitively established on imaging. (C) Coronal reformat confirms the craniocaudal extent of the mass and its relationship to adjacent bowel loops. No pneumoperitoneum or significant free intraperitoneal fluid is evident

Abdominal computed tomography (CT) demonstrated a heterogeneous 7 cm mass in the right hemiabdomen with central hypoattenuation, irregular peripheral enhancement, and marked surrounding inflammatory fat stranding, abutting the ascending colon/hepatic flexure and the second portion of the duodenum. The pericecal location and appendiceal enlargement favored, but did not definitively establish, an appendiceal origin on imaging; no discrete transition zone or small-bowel-feces sign was identified, and there was no pneumoperitoneum or significant free intraperitoneal fluid (Figure 1).

**Figure 2:**
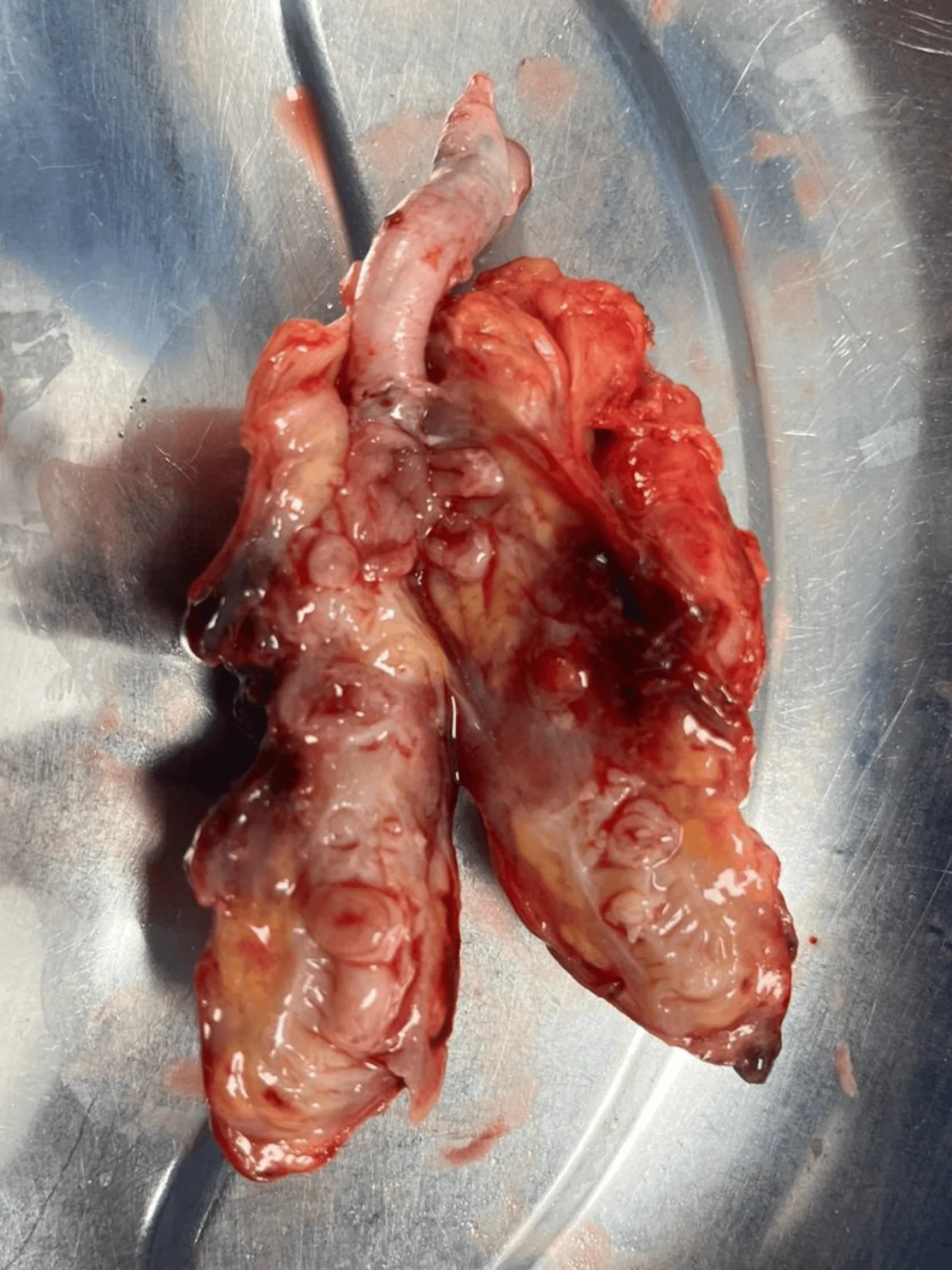
Gross appearance of the excised appendiceal specimen Appendix measuring 5 cm in length and 1.5 cm in diameter, surrounded by multilobulated periappendiceal soft tissue. Diffuse appendiceal wall thickening and adjacent multilobulated periappendiceal tissue imparted a nodular gross appearance to the specimen

Given these findings and the patient's clinical status, the decision was made to proceed directly to open exploratory laparotomy rather than a laparoscopic approach. Following induction of general anesthesia, an exploratory midline laparotomy was performed to ensure adequate exposure and allow atraumatic handling of the mass, given its considerable size and adhesion to the terminal ileum. The appendix showed diffuse wall thickening, and adjacent multilobulated periappendiceal tissue imparted a nodular gross appearance to the specimen, which was filled with gelatinous mucoid contents. The cecum and the appendiceal base were carefully inspected and showed no gross abnormality. Adhesive bands involving the terminal ileum were meticulously lysed by sharp dissection; the ileum was confirmed viable, with no evidence of ischemia or serosal injury after adhesiolysis. The appendiceal mass and adherent periappendiceal tissue were excised en bloc, achieving total excision of the lesion while preserving the integrity of the small bowel and avoiding iatrogenic rupture (Figure 2).

Total operative time was one hour and 39 minutes, with an estimated blood loss of 200 mL. After careful inspection confirmed no further pathology or mucin contamination, the peritoneal cavity was copiously irrigated, and the incision closed in layers. Histopathological examination confirmed a LAMN with acute periappendicitis. The appendiceal lumen was occupied by mucin with intraluminal calcifications. No invasion of the lamina propria, lymphovascular invasion, or perineural invasion was identified, and the circumferential and basal surgical margins were negative for neoplasia. Fibrinopurulent peritonitis was noted. The lesion was staged as pTis(LAMN) according to the tumor, node, and metastasis (TNM) classification. Immunohistochemistry was not performed, as the institution did not cover this ancillary study and would have required an out-of-pocket cost that the patient was unable to afford.

The patient tolerated oral intake at 48 hours postoperatively and was discharged in good condition on postoperative day 3 (72 hours). The publication of this case report, including accompanying images, was reviewed and approved by the institutional ethics committee, and written informed consent for publication was obtained from the patient. Follow-up included a postoperative visit at two weeks and outpatient clinical review at three months; double-contrast CT at six months and joint follow-up with oncologic surgery were planned as part of the surveillance strategy. A summary of the findings is shown in Table 1.

**Table 1:** Summary of clinical, laboratory, imaging, operative, and histopathological finding AJCC: The American Joint Committee on Cancer; MCV: mean corpuscular volume; MCH: mean corpuscular hemoglobin; CRP: C-reactive protein; LAMN: low-grade appendiceal mucinous neoplasm

Category	Finding
Age/sex	53 years/male
Presentation	4-day history of periumbilical pain, obstipation, nausea, bilious vomiting
Exam	Abdominal distension, decreased bowel sounds, rebound tenderness; tachycardia, mild diaphoresis
Hemoglobin/hematocrit	15.6 g/dL (13-18)/45.4% (40-54): normal
MCV/MCH	86 fL (80-100)/29.5 pg (27-32): normal
Platelets	127,000/µL (130,000-450,000): mild thrombocytopenia
Leukocytes	9,750/µL (4,000-10,000): normal
Neutrophils/lymphocytes	82.4% (50-65): neutrophilia/11.6% (20-40): relative lymphopenia
Eosinophils/basophils/monocytes	0.70% (1-5)/0.2% (0-2)/5% (4-10)
Glucose	154 mg/dL (70-105): hyperglycemia
CRP/electrolytes/creatinine/lactate/tumor markers	Not obtained
Imaging	CT: 7 cm heterogeneous right hemiabdominal mass, pericecal fat stranding, favored but did not confirm appendiceal origin; no discrete transition zone or small-bowel-feces sign identified; no pneumoperitoneum or significant free fluid
Operative approach	Open exploratory laparotomy (chosen given the patient's clinical condition)
Operative time/blood loss	1 h 39 min/200 mL
Operative findings	Dilated, mucin-filled appendix (5 × 1.5 cm) densely adherent to terminal ileum; cecum and appendiceal base grossly normal on inspection; terminal ileum viable after adhesiolysis; en bloc adhesiolysis and appendectomy without rupture
Histopathology	LAMN with acute periappendicitis; no lamina propria, lymphovascular, or perineural invasion; circumferential and basal margins negative; fibrinopurulent peritonitis; lumen occupied by mucin with intraluminal calcifications
Staging	pTis(LAMN), AJCC 8th edition
Immunohistochemistry	Not performed (not covered by the institution; cost not affordable for the patient)
Postoperative course	Oral intake at 48 h; discharge on postoperative day 3 (72 h)
Follow-up plan	Postoperative visit at 2 weeks; outpatient review at 3 months; double-contrast CT at 6 months; joint follow-up with oncologic surgery

## Discussion

This case reinforces the need to maintain a high index of suspicion for underlying appendiceal pathology when obstructive symptoms persist despite the absence of a palpable mass. The differential diagnosis of a periappendiceal inflammatory mass with obstructive symptoms is broad [12,13].

Acute appendicitis and periappendiceal abscess typically present with a shorter symptom course and a homogeneously inflamed, noncystic appendix on imaging, in contrast to the cystic, mucin-distended appendix seen in LAMN [8]. Cecal carcinoma and invasive appendiceal mucinous adenocarcinoma may produce a similar obstructive picture but are distinguished from LAMN by evidence of wall invasion, irregular infiltrative margins, or regional lymphadenopathy on imaging and, definitively, by invasion beyond the muscularis propria on histopathology [1]. A nonneoplastic appendiceal mucocele can mimic LAMN radiologically but lacks the epithelial dysplasia that defines a neoplastic process. Cecal or ileocecal Crohn's disease may present with a right-sided inflammatory mass and obstruction but is usually accompanied by a longer symptomatic history and bowel wall thickening over a longer segment. Internal hernia, as reported in other LAMN-obstruction cases [8], produces obstruction through bowel entrapment within a peritoneal or mesenteric defect rather than external compression from an adherent mass, as in the present case. In our patient, the combination of a cystic, mucin-filled appendiceal mass on CT and dense, noninvasive adhesion to the terminal ileum at laparotomy favored LAMN over these alternatives, a diagnosis subsequently confirmed on histopathology.

CT remains the preoperative gold standard, typically revealing a tubular or cystic structure in the right iliac fossa with periappendiceal fat stranding, a finding suggestive of mucocele; as in the present case, the precise organ of origin is not always definitively established preoperatively, and diagnosis is ultimately confirmed intraoperatively and histopathologically. Mural calcifications and luminal fluid density should be interpreted carefully, as these features often point to a mucinous rather than purely inflammatory etiology [1,6]. In patients with intestinal obstruction and a transition point in the right lower quadrant, a high index of suspicion for an appendiceal etiology is warranted even when, as in this case, no discrete transition zone or small-bowel-feces sign is identified [7].

A review of the available literature identifies only a small number of case reports describing mechanical small bowel obstruction as the presenting feature of LAMN, most attributing obstruction to an adhesive band or internal hernia between the lesion and adjacent small bowel rather than to direct dense adhesion from an intact mass, as observed in our patient [7-9]. This scarcity reinforces that obstruction is not a typical manifestation of LAMN and supports its inclusion in the differential diagnosis of unexplained bowel obstruction even in patients without a palpable mass or prior abdominal surgery (Table 2).

**Table 2:** Comparison with previously reported cases of LAMN presenting with mechanical obstruction LAMN: low-grade appendiceal mucinous neoplasm

Case	Age/sex	Presentation	Mechanism of obstruction	Surgical management	Outcome
Present case	53 M	4-day periumbilical pain, obstipation, bilious vomiting	Dense adhesion of an intact LAMN to the terminal ileum	Open adhesiolysis + en bloc appendectomy	Uneventful; pTis(LAMN); surveillance ongoing
Gupta et al., 2023 [7]	67 F	2-day constipation, periumbilical pain, bilious vomiting	Adhesive band from appendix to ileum forming a closed loop with distal ileal gangrene	Ileocecal resection + ileocolic anastomosis	LAMN confirmed on histopathology
Karrabi, 2026 [8]	72 M	4-day intermittent abdominal pain, nausea, vomiting, obstipation	Internal hernia at the site of a LAMN at the appendiceal tip	Appendectomy + mesenteric mass excision + hernia repair	No recurrence at 6-month follow-up
Lin et al., 2022 [9]	84 F	Recurrent obstructive episodes over 3 months, initially managed conservatively	Tumor directly adherent to/invading the ileum with a herniated bowel segment	Emergent surgical resection	LAMN confirmed on histopathology

Histopathological confirmation of LAMN requires careful distinction from a simple inflammatory mucocele, since the former carries a distinct risk of peritoneal dissemination [14]. Given the potential for occult malignancy and the risk of pseudomyxoma peritonei through iatrogenic or spontaneous rupture, management must prioritize atraumatic handling and complete surgical excision. In this patient, appendectomy alone, rather than ileocecal resection, was considered adequate because the terminal ileum, although densely adherent, was confirmed viable with no evidence of ischemia or invasion after careful adhesiolysis; the cecum and appendiceal base were grossly uninvolved; and final histopathology confirmed negative margins with no lymphovascular, perineural, or lamina propria invasion.

Immunohistochemistry, while not required for the diagnosis of a typical LAMN confined to the appendix, plays a defined role when the lesion is discovered at an ectopic site or when the primary origin of a mucinous tumor is uncertain. LAMN epithelium is characteristically CK20-positive and CDX-2-positive, reflecting an intestinal/appendiceal immunophenotype, while co-expression of CK7 and villin further supports gastrointestinal differentiation. This panel is particularly useful in distinguishing a primary appendiceal mucinous neoplasm with secondary ovarian, peritoneal, or endometrial involvement from a primary ovarian mucinous tumor or a colorectal mucinous adenocarcinoma secondarily involving the appendix. In the present case, the diagnosis was established on hematoxylin and eosin morphology alone, including confirmation of negative margins and absence of lamina propria, lymphovascular, and perineural invasion; immunohistochemistry was not performed because it was not covered by the institution and represented an out-of-pocket cost the patient could not afford. We report this openly as a resource-related limitation rather than a diagnostic gap, since the morphological criteria for LAMN were unambiguous in this specimen.

Long-term outcomes for LAMN are generally favorable, although the risk of late progression to pseudomyxoma peritonei warrants continued surveillance. There is no universal consensus on the optimal frequency or duration of follow-up, but current practice typically includes periodic clinical evaluation, serial tumor-marker monitoring, and cross-sectional imaging to detect recurrent or intraperitoneal mucinous disease [4,8]. In this patient, surveillance was structured around a two-week postoperative visit, a three-month clinical review, and a planned double-contrast CT at six months, with joint follow-up by oncologic surgery.

Limitations

This report has several limitations. Serum C-reactive protein, electrolytes, creatinine, and lactate were not obtained, limiting objective assessment of the systemic inflammatory and metabolic response at presentation. Immunohistochemistry was not performed for the reasons described above. Digitized histopathology slide images were not available in the institutional record and could therefore not be included as a figure, and the original CT images could not be re-exported at higher resolution or with structure-specific annotation. These limitations reflect resource constraints at the treating institution rather than gaps in clinical management and are disclosed transparently to allow appropriate interpretation of the case.

## Conclusions

This case illustrates that LAMN, although rare and typically indolent, can present as mechanical small bowel obstruction through dense adhesion to the terminal ileum in the absence of perforation, torsion, or internal herniation, a mechanism only sporadically reported in the literature and the principal educational contribution of this report. Preoperative CT raised suspicion of an appendiceal mass and allowed the surgical team to plan an atraumatic, en bloc resection, but the diagnosis itself was established only on histopathological examination, which confirmed negative margins and no invasive features (pTis(LAMN), AJCC eighth edition). Meticulous intraoperative handling to avoid iatrogenic rupture remains the cornerstone of management given the risk of pseudomyxoma peritonei. The patient's surveillance plan reflects the approach adopted for this individual case; given the limited number of comparable reports, we refrain from broader surveillance recommendations beyond what was carried out here.
